# Prevalence of respiratory viruses among paediatric patients in acute respiratory illnesses in Malaysia

**DOI:** 10.1371/journal.pone.0265288

**Published:** 2022-08-03

**Authors:** Yoke Lee Low, Shin Yee Wong, Eric Kim Hor Lee, Mohd Hareeff Muhammed

**Affiliations:** 1 Pantai Premier Pathology Sdn Bhd, Kuala Lumpur, Malaysia; 2 Pantai Hospital Kuala Lumpur, Kuala Lumpur, Malaysia; Health Directorate, LUXEMBOURG

## Abstract

**Objectives:**

Acute respiratory infections (ARIs) are one of the leading causes of childhood morbidity and mortality worldwide. However, there is limited surveillance data on the epidemiological burden of respiratory pathogens in tropical countries like Malaysia. This study aims to estimate the prevalence of respiratory pathogens causing ARIs among children aged <18 years old in Malaysia and their epidemiological characteristics.

**Methods:**

Nasopharyngeal swab specimens received at 12 laboratories located in different states of Malaysia from 2015–2019 were studied. Detection of 18 respiratory pathogens were performed using multiplex PCR.

**Results:**

Data from a total of 23,306 paediatric patients who presented with ARI over a five-year period was studied. Of these, 18538 (79.5%) were tested positive. The most prevalent respiratory pathogens detected in this study were enterovirus/ rhinovirus (6837/ 23000; 29.7%), influenza virus (5176/ 23000; 22.5%) and respiratory syncytial virus (RSV) (3652/ 23000; 15.9%). Throughout the study period, RSV demonstrated the most pronounce seasonality; peak infection occurred during July to September. Whereas the influenza virus was detected year-round in Malaysia. No seasonal variation was noted in other respiratory pathogens. The risk of RSV hospitalisation was found to be significantly higher in children aged less than two years old, whereas hospitalisation rates for the influenza virus peaked at children aged between 3–6 years old.

**Conclusion:**

This study provides insight into the epidemiology and the seasonality of the causative pathogens of ARI among the paediatric population in Malaysia. Knowledge of seasonal respiratory pathogens epidemiological dynamics will facilitate the identification of a target window for vaccination.

## Introduction

Acute respiratory infections (ARIs) are one of the leading causes of childhood morbidity and mortality worldwide. It is estimated that each year more than 2 million children die from ARIs with 70% of the mortality occurring in Africa and Southeast Asia [1]. Most ARIs are of viral origin in children less than 5 years old [2]. The association between ARIs and different viral pathogens ranges between 40% to 90% [3–6]. Influenza A and B virus, enterovirus/ rhinovirus, respiratory syncytial virus (RSV), adenovirus and parainfluenza virus are among the common pathogens detected [7, 8]. With more and more new pathogens like human metapneumovirus (hMPV) and human bocavirus reported over the decades [9], there is an increased urgency to study the epidemiology of respiratory tract pathogens in order to facilitate the planning of appropriate disease prevention and control strategies.

Detection of the specific respiratory viral pathogens in ARIs is essential for the proper management of patients and to avoid the unnecessary use of antibiotics [10]. Over the past decades, culture sensitivity, and serology testing has been the mainstay of clinical laboratory diagnosis for respiratory pathogen detection. Increasing use of molecular techniques including polymerase chain reaction (PCR) has improved the sensitivity of detecting and identifying respiratory pathogens, providing new information on the epidemiology of ARIs besides contributing to a better understanding of the seasonality of the causative pathogens and their association with certain clinical manifestations [11, 12].

Respiratory syncytial virus (RSV) and influenza virus are recognised as the most significant causes of ARIs in children worldwide [13]. Nearly all children are infected with RSV at least once during the first two years of life, with approximately two-third being infected by the end of their first year. The severity of RSV infections decreases with age, with a mild-to-moderate upper respiratory tract infection in healthy adults. [14]. Unlike RSV, influenza can affect people in any age group. Influenza complications and hospitalisation however are more common in childhood, especially in children age < 5 years, adults aged ≥ 65 years and those with chronic disease [15, 16]. In most temperate climates, influenza and RSV activity consistently peaked during winter months while the timing of epidemics was found to be more diverse in the tropics [17].

Despite the burden of ARIs on morbidity and mortality in children, only limited data is available from countries in tropical and subtropical areas [18–20]. In order to determine the ongoing burden of viral-ARIs in the Malaysian paediatric population, a more representative surveillance data is needed. This will help in the implementation of prevention and treatment strategies for the management of ARIs in children. Thus, our study is aimed at evaluating the epidemiology of respiratory pathogens causing ARIs among children aged < 18 years in Malaysia, a tropical country north of equator. We further characterise the seasonal pattern and age-specific differences in influenza and RSV activity.

## Methods

### Subject enrolment and sampling

This is a retrospective observational study to evaluate the overall viral incidence among Malaysian paediatric patients using the database in Pantai Premier Pathology, Kuala Lumpur.

Pantai Premier Pathology is one of the established and accredited private medical laboratories with several branches located in various states in Malaysia. Nasopharyngeal/ nasal swab specimens received at 12 different laboratories located in different states of Malaysia from January 2015 to December 2019 were analysed. Swab samples were collected from paediatric in- and out-patients (age ≤18) who presented with signs and symptoms of upper or/and lower respiratory tract infections including runny or stuffy nose, sore throat, cough, chills, fatigue and muscle ache. Nasopharyngeal specimens were obtained from these patients according to standard technique and placed in viral transport media. The requests for multiplex PCR testing were at the discretion of the physicians.

### Ethical statement

Ethic approval for this study was obtained from Pantai Hospital Kuala Lumpur Research and Ethics Committee (Ethics Approval Number: PHKL-EC-2019-0012).

### Multiplex polymerase chain reaction

Nasopharyngeal/ nasal samples were analysed by multiplex PCR method using Luminex NxTAG respiratory pathogen panel (Luminex Molecular Diagnostics, Toronto), BioFire FilmArray respiratory panel RP, RP2 (BioFire Diagnostics, Utah) or QIAstat-Dx respiratory panel V2 (Qiagen, Hilden) according to the manufacturer’s instructions. 4 pathogens were excluded from the analysis due to inherent differences in the reagent kits used. These include bocavirus, *Bordetella pertussis*, *Chlamydophila pneumoniae* and MERS coronavirus. 18 respiratory virus subtypes including non-specific influenza A, H1 subtype, H1N1(2009) subtype, H3 subtype, influenza B, parainfluenza1, parainfluenza2, parainfluenza3, parainfluenza4, coronavirus NL63, coronavirus HKU1, coronavirus 229E, coronavirus OC43, human metapneumovirus (hMPV), adenovirus, enterovirus/ rhinovirus, respiratory syncytial virus and one bacterial *Mycoplasma pneumoniae* were analysed. When more than one virus was detected simultaneously from the same patient, the findings will be recorded as multiple infections.

### Data collection

Demographic data including medical record number, age and gender, respiratory sample results including the type of virus and the date of sample collection were retrieved from the laboratory database. Data were cleaned, screened from errors and duplication prior to data analysis. Patients who were found to have more than 1 positive specimens within the same week were excluded from the final analysis.

### Statistical analysis

Data was analysed with Statistical Package for Social Science (SPSS) version 26.0. Descriptive statistics (e.g frequency and percentage) were used for demographic characteristics. ANOVA and Kruskal-Wallis test is used to analyse non-normal distributed data. A *p*-value <0.05 was considered statistically significant.

## Results

### Demographic characteristics of study population

During the five-year period from 2015–2019, specimens from a total of 23,306 paediatric patients who presented with ARI were analysed. Table 1 outlines the social-demographic variables of all the sample population. On average, 51.9% of the patients were male. The mean (±SD) age for the study population was 2.9 (±3.1) years with the majority of the patients (36.1%) in the age group of 1–2 years old. 5391 (23.1%) of the patients were less than one year old, 8418 (36.1%) were between 1–2 years old, 6763 (29.0%) were 3–6 years old and 2734 (11.7%) were more than 7 years old. The numbers of samples received at the laboratory have increased 5-fold in 5 years with the compounded annual growth rate (CAGR) of 50%, from 1680 in 2015 to 8520 in 2019.

**Table 1:** Demographic data of the paediatric population analysed from year 2015 to 2019.

	2015	2016	2017	2018	2019	Total
**Gender**						
Male	912 (54.3%)	1402 (55.5%)	2221 (55.6%)	3650 (55.4%)	3904 (45.8%)	12089 (51.9%)
Female	768 (45.7%)	1124 (44.5%)	1773 (44.4%)	2936 (44.6%)	4616 (54.2%)	11217 (48.1%)
**Age**						
<1	449 (26.7%)	681 (27.0%)	1043 (26.1%)	1435 (21.8%)	1783 (20.9%)	5391 (23.1%)
1–2	570 (33.9%)	909 (36.0%)	1512 (37.9%)	2384 (36.2%)	3043 (35.7%)	8418 (36.1%)
3–6	441 (26.3%)	666 (26.4%)	1078 (27.0%)	2013 (30.6%)	2565 (30.1%)	6763 (29.0%)
7–12	189 (11.3%)	240 (9.5%)	324 (8.1%)	668 (10.1%)	908 (10.7%)	2329 (10.0%)
13–17	31 (1.8%)	30 (1.2%)	37 (0.9%)	86 (1.3%)	221 (2.6%)	405 (1.7%)
Total	1680 (100.0%)	2526 (100.0%)	3994 (100.0%)	6586 (100.0%)	8520 (100.0%)	23306 (100.0%)

### Prevalence of respiratory pathogens

Of the 23,306 swab specimens received, 18538 (79.5%) were tested positive. Notably the total number of pathogens detected increased gradually from 1452 in year 2015 to 8965 in year 2019 (Table 2). A total of 23000 pathogens were detected by multiplex PCR. 5 predominant respiratory pathogens detected in this study were enterovirus/ rhinovirus (6837/ 23000; 29.7%), followed by influenza virus (5176/ 23000; 22.5%), RSV (3652/ 23000; 15.9%), adenovirus (2637/ 23000; 11.5%), and parainfluenza virus (2333/ 23000; 10.1%); Other less frequently detected respiratory pathogens including hMPV (819/ 23000; 3.6%) and *Mycoplasma pneumoniae* (338/ 23000; 1.5%).

**Table 2:** Prevalence of respiratory pathogens detected from year 2015 to 2019.

	2015	2016	2017	2018	2019	Total
Adenovirus	234 (16.1%)	190 (9.1%)	479 (12.2%)	918 (14.0%)	816 (9.1%)	2637 (11.5%)
Coronavirus	39 (2.7%)	73 (3.5%)	182 (4.6%)	195 (3.0%)	330 (3.7%)	819 (3.6%)
Human Metapneumovirus	117 (8.1%)	128 (6.1%)	279 (7.1%)	302 (4.6%)	382 (4.3%)	1208 (5.3%)
Enterovirus/ Rhinovirus	465 (32.0%)	579 (27.7%)	1037 (26.4%)	1968 (30.0%)	2788 (31.1%)	6837 (29.7%)
Influenza A	178 (12.3%)	166 (7.9%)	608 (15.5%)	833 (12.7%)	1374 (15.3%)	3159 (13.7%)
Influenza B	38 (2.6%)	250 (12.0%)	269 (6.9%)	620 (9.4%)	840 (9.4%)	2017 (8.8%)
Mycoplasma pneumoniae	0 (0%)	21 (1.0%)	34 (0.9%)	79 (1.2%)	204 (2.3%)	338 (1.5%)
Parainfluenza	118 (8.1%)	184 (8.8%)	365 (9.3%)	678 (10.3%)	988 (11.0%)	2333 (10.1%)
Respiratory Syncytial Virus	263 (18.1%)	500 (23.9%)	672 (17.1%)	974 (14.8%)	1243 (13.9%)	3652 (15.9%)
**Total**	1452 (100.0%)	2091 (100.0%)	3925 (100.0%)	6567 (100.0%)	8965 (100.0%)	23000 (100.0%)

### Percentage positivity of pathogens detected in different paediatric age groups

**Fig 1:**
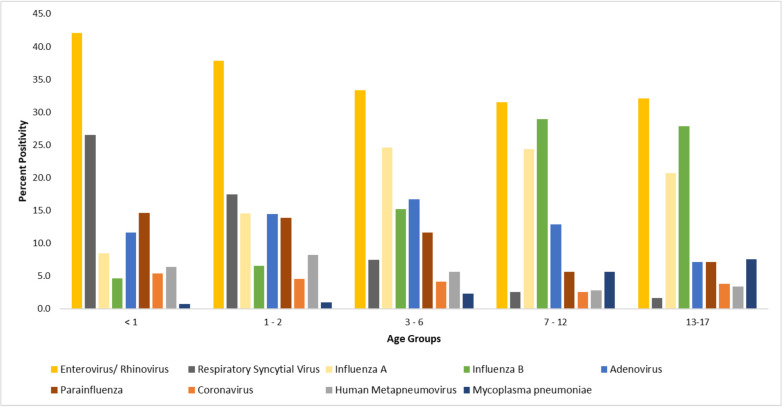
The percentage positivity of respiratory pathogens detected in different paediatric age groups.

Patients were divided into five different age groups namely, <1, 1–2, 3–6, 7–12 and 13–17 years old (Fig 1). Two respiratory pathogens found commonly in children below one year old (p-value <0.001) with the prevalence of 42.1% and 26.6% for enterovirus/ rhinovirus and RSV, respectively. On the other hand, influenza virus (peaked at 7–12 years old, 53.3%), *Mycoplasma pneumoniae* (peaked at 13–17 years old, 7.2%) were more prevalent in older age groups (p-value <0.001). Prevalence of other respiratory pathogens peaked at different age groups: adenovirus (3–6 years old, 16.7%), coronavirus (<1 year old, 5.4%), hMPV (1–2 years old, 8.2%) and parainfluenza virus (<1 year old, 14.6%).

### Co-infections detected in the samples

**Fig 2:**
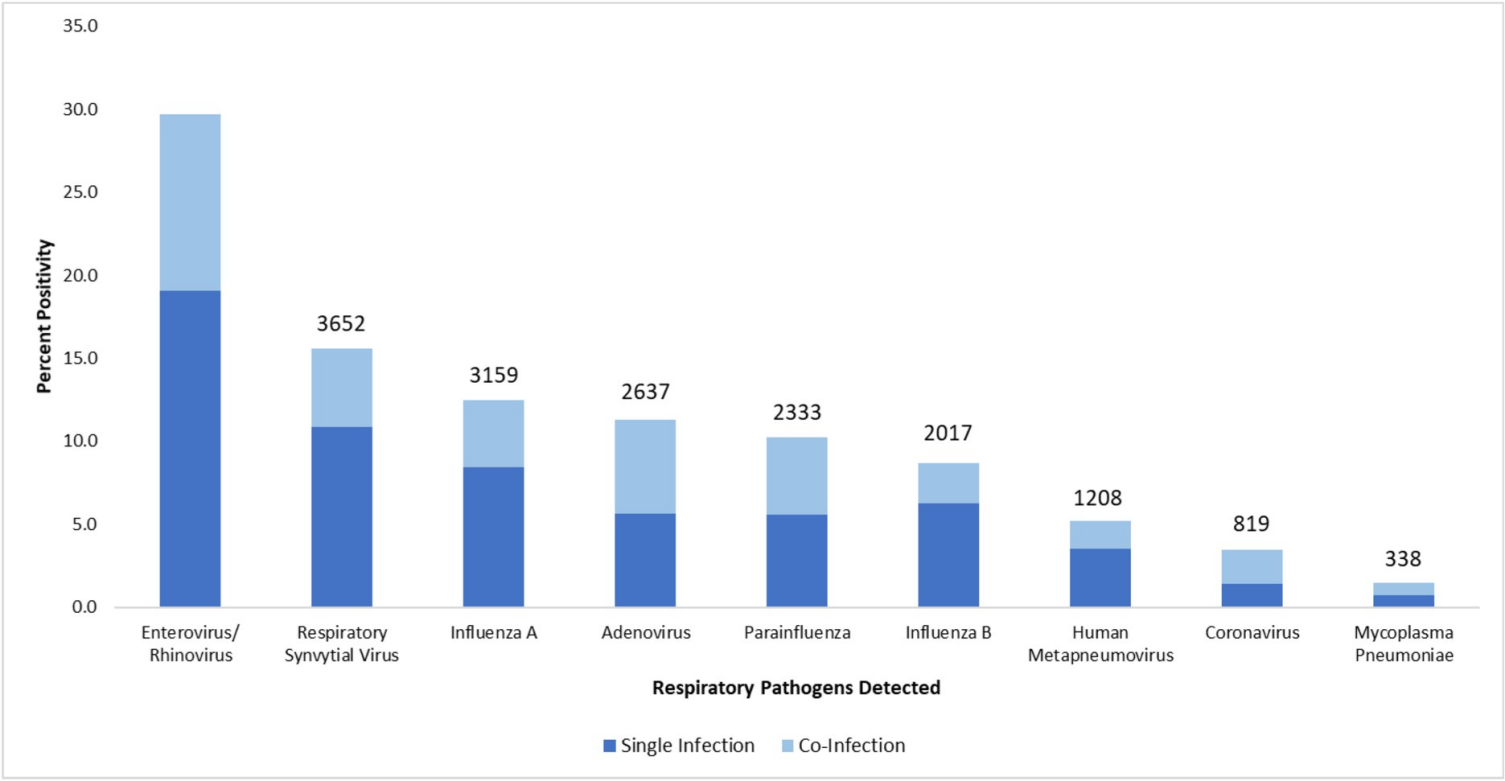
Percentage positivity of single and co-infections detected in each respiratory pathogen.

Among the respiratory pathogens, enterovirus/ rhinovirus (76.8%), influenza B (72.1%) and RSV (69.5%) were predominantly detected as a single pathogen. Whereas coronavirus (59.6%) and Adenovirus (50.3%) were more commonly detected as co-infections. Co-infections were detected in 4242 samples (Fig 2), with a detection rate of 18.2% (4224/ 23306) of total specimens and 22.9% (4242/18538) of positive samples observed. Co-infections were found to be most prevalent in children less than 1 years old (20.0%), while it was least common in elder children age 13–17 years old (7.2%). This difference was statistically significant (p-value <0.001).

### Seasonal activity of respiratory pathogens

**Fig 3:**
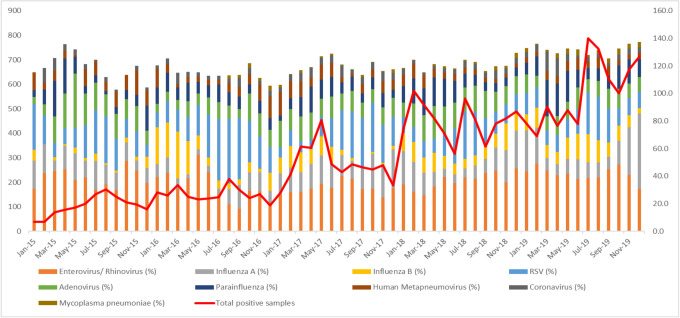
Monthly distribution of respiratory pathogens detected from January 2015 to December 2019.

**Fig 4:**
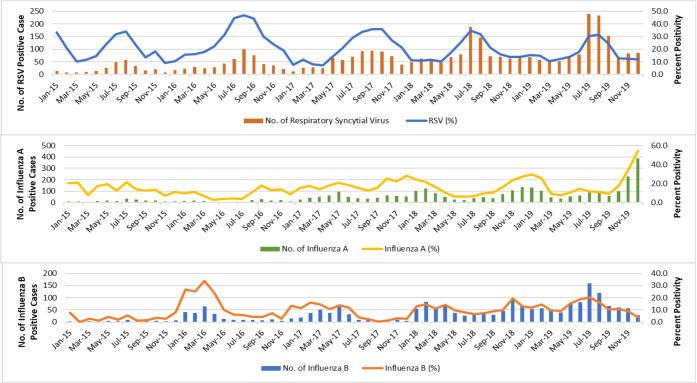
Monthly distribution of influenza A, influenza B and respiratory syncytial virus from January 2015 to December 2019.

The seasonal distribution of the respiratory pathogens from January 2015 to December 2019 is shown in Fig 3. Overall positivity detection rate increased gradually year by year. Respiratory pathogens were detected throughout the year. Throughout the five-year study period, RSV demonstrated the most pronounced seasonality, with peak infection occurring from July to September (Fig 4). No seasonal variation was noted for influenza, enterovirus/ rhinovirus, adenovirus, coronavirus, parainfluenza virus, hMPV and *Mycoplasma Pneumoniae*. These viruses are detected year-round in Malaysia.

### Age-dependent distribution of RSV and influenza infections

RSV (1390/5391) was predominantly detected in children less than 1-year old [P = <0.001, RR = 2.05 (1.94–2.18), OR = 2.42 (2.24–2.61)] (Table 3). On the contrary, influenza A H1N1 [P = <0.001, RR = 0.42 (0.36–0.48), OR = 0.39 (0.34–0.46)], influenza A H3 [P = <0.001, RR = 0.39 (0.31–0.49), OR = 0.38 (0.31–0.48)], non-specific influenza A [P = <0.001, RR = 0.43 (0.32–0.59), OR = 0.43 (0.31–0.58)] and influenza B [P = <0.001, RR = 0.36 (0.32–0.42), OR = 0.34 (0.29–0.39)] were found to be more prevalent in children ≥1-year old (Table 3).

**Table 3:** Distribution of influenza A, influenza B and respiratory syncytial virus in different age group.

Pathogens	< 1 year old (n = 5391)	≥ 1 years old (n = 17915)	P-value	Risk Ratio (RR) [95% CI]	Odds Ratio (OR) [95% CI]
Influenza A H1N1	207 (3.8%)	1652 (9.2%)	< 0.001	0.42 [0.36–0.48]	0.39 [0.34–0.46]
Influenza A H1	21 (0.4%)	53 (0.3%)	0.284	1.32 [0.80–2.20]	1.32 [0.80–2.20]
Influenza A H3	87 (1.6%)	738 (4.1%)	< 0.001	0.39 [0.31–0.49]	0.38 [0.31–0.48]
Non-Specific Influenza A	46 (0.9%)	355 (2.0%)	< 0.001	0.43 [0.32–0.59]	0.43 [0.31–0.58]
Influenza B	199 (3.7%)	1818 (10.1%)	< 0.001	0.36 [0.32–0.42]	0.34[0.29–0.39]
Respiratory Syncytial Virus	1390 (25.8%)	2249 (12.6%)	< 0.001	2.05 [1.94–2.18]	2.42 [2.24–2.61]

### Age-specific hospitalisation rates in influenza and RSV

**Fig 5:**
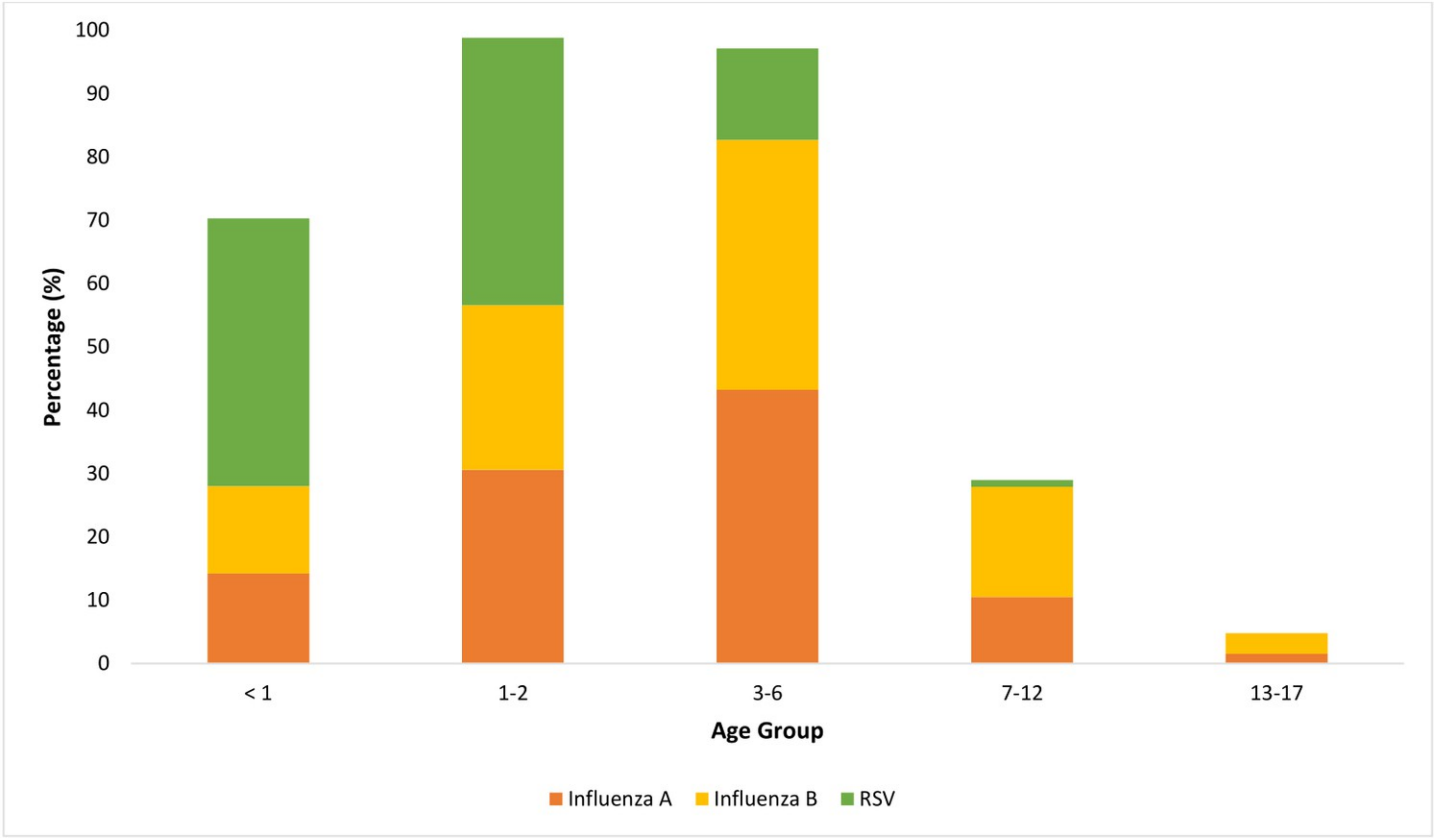
Percentage total hospitalisation attributable to RSV and influenza viruses.

The risk of RSV hospitalisation was found to be significantly higher in children aged less than 2 years old; 42.3% in children less than 1 year old and 42.2% in children 1–2 years old respectively (Fig 5). RSV hospitalisation rates decreased with increasing age and did not contribute to any hospitalisations in children age group 13–17 years old. Influenza A and B share a similar pattern where hospitalisation rate was found to be more prevalent in children less than 6 years old, with the peak at 3–6 years old (influenza A = 43.2%, influenza B = 39.5%).

## Discussion

The Malaysian Ministry of Health reports that disease in the respiratory system is one of the major causes of hospitalisation in 2018, 13.86% and 18.73% in government and private hospitals, respectively [21]. However, there is limited epidemiological data as to the causative pathogens available in tropical countries, like Malaysia. This could be partly attributed to a lack of diagnostic capacity for viral detection. Previous studies using immunofluorescence (IF) and viral cultures had poor viral detection rates thus considerably underestimating the true burden of viral respiratory infections in Malaysia [22, 23]. Recent studies in Malaysia shown that real-time PCR offers a more advanced application, allowing the detection of a variety of viral and bacterial pathogens within hours [24, 25]. Rapid identification of the causative pathogens would aid in the timely initiation and management of ARIs [11, 26].

18 respiratory virus subtypes including coronavirus and human metapneumovirus were included. Of 23,306 swab specimens received, a total of 23,000 pathogens including co-infections were detected by multiplex PCR method. 5 main respiratory pathogens detected in this study was enterovirus/ rhinovirus (29.7%), followed by RSV (15.9%), influenza A (13.7%), adenovirus (11.5%), and parainfluenza virus (10.1%). Noted PCR testing for respiratory pathogens grow at a higher CAGR compared to rapid influenza diagnostic testing, mainly due to higher awareness among the clinicians on the advantage of PCR testing.

Both enterovirus and rhinovirus are within the *Picornaviridae* family. Due to their common genomic features and their high sequence homology, both viruses unfortunately cannot be differentiated by this methodology. Data showed that enterovirus/ rhinovirus was the most predominant pathogens in paediatric ARIs. This is consistent with Yew SM et al. study that showed enterovirus/rhinovirus as the most common virus in the paediatric population, especially among infants (less than 12 months old) [25]. Our study showed that enterovirus/ rhinovirus was detected in 42.1% of the children less than one year old. This finding is consistent with other studies where enterovirus/ rhinovirus are two to three times more frequently detected in infants than older children [26].

This study showed that as the age of the paediatric patients increased, the positive detection rate decreased proportionately from 78% in the age group less than one year old to 58% in the older children group, age 13–17 years old. Studies showed that young children are prone to respiratory infections as their natural passive immunity from the mother diminishes within months after birth and immunological protection will eventually be acquired through infection or from immunisation [27].

Multiple infections were observed in 18.9% of the samples with at least two respiratory pathogens. The significance of co-infections in this study could not be determined as we did not have information on its clinical severity. Some studies have suggested that multiple infections were associated with lengthened duration of hospitalisation, intensive care unit admission, and prolonged mechanical ventilation support and death [28], whereas other studies do not show any differences in severity between single and multiple respiratory infections [29].

The data obtained showed shown that respiratory pathogens were detected year-round. RSV demonstrated the most pronounced seasonality, with peak infection occurring from during July to September. This is supported by a few studies in the region [30–33]. Thongpan et al. in a recent study discovered that RSV infections happened predominantly in the second half of the year between July to November, likely due to the rainy season [30]. Singapore observed similar seasonality patterns with a slight increase in RSV infections between the months of June and August annually [31]. A high proportion of RSV-positive children (25.8%) were observed in infants less than one year old. RSV infected children below 2 years old had a high hospitalisation rate (84.5%). It is also the leading cause of hospitalisation in children less than 2 years old worldwide [32], causing severe pneumonia and bronchiolitis and is associated with a higher risk of developing asthma and recurrent wheezing later in life [33]. Influenza infection from our study, however, was present year-round with no annual seasonality observed. Influenza displays a distinct seasonal pattern in temperate countries, with annual epidemic occurring in the winter [34]. This seasonal pattern however is less commonly reported in tropical or subtropical areas showing year-round circulation with unpredictable variation in intensity [35, 36]. Influenza related hospitalisation rate was found to be more prevalent in children less than 6 years old in our study. A similar pattern was also observed where the age-specific hospitalisation rate was higher in younger children less than 5 years old [37].

Given the high RSV disease burden in our younger population, the development of an effective RSV vaccine is of priority. Currently, there is no licensed vaccine for RSV. Several clinical trials are in various stages of development to assess the safety and effectiveness of different RSV vaccine candidates [38]. Understanding the RSV epidemiological patterns helps determine the optimal timing for vaccination. Surveillance of RSV has been integrated into the existing influenza surveillance system in several high-income countries. However, most low- and middle-income countries where the RSV burden is likely to be the greatest has limited or no national data [39]. To our knowledge, this is one of the largest sample sizes of laboratory confirmed influenza and RSV cases in Malaysia.

The US Advisory Committee on Immunisation Practices (ACIP) recommends annual influenza vaccination starting from a minimum age of 6 months to reduce influenza-associated morbidity and mortality [40]. Various studies including clinical trials and observational studies have consistently shown that influenza vaccines are safe [41]. However, the complex seasonal patterns of influenza activity in this region have complicated the determination of an optimal timing for influenza vaccination. Our study shows a high rate of influenza infection hospitalisation in children below 6 years of age, with a peak in the 3–6 years old age group. In view of this finding, targeting influenza vaccination in children below 6 years old should be considered.

There are two main limitations in this study. As the data collected was retrospectively and originated from our network of laboratories, the severity and associated comorbidities of the patients could not be assessed. As such, we were also unable to determine the clinical significance of respiratory co-infections. Also, we are unable to attribute the meteorological factors (e.g., temperature, rain and humidity) with the respiratory pathogens pattern. Despite these limitations, the strength of this study is the large sample size providing an insight into the prevalence of respiratory pathogens amongst Malaysian paediatric population. Knowledge of the respiratory pathogens epidemiological dynamics will facilitate the identification of a target age and an ideal window for vaccination.

## Supporting information

S1 Data (XLSX)
